# A DFT Study of the Geometrical, Spectroscopical and Reactivity Properties of Diindolylmethane-Phenylboronic Acid Hybrids

**DOI:** 10.3390/molecules22101744

**Published:** 2017-10-17

**Authors:** Amira Jalil Fragoso-Medina, René Gerardo Escobedo-González, María Inés Nicolás-Vázquez, Gabriel Arturo Arroyo-Razo, María Olivia Noguez-Córdova, René Miranda-Ruvalcaba

**Affiliations:** Departamento de Ciencias Químicas, Facultad de Estudios Superiores Cuautitlán, Campo 1, Universidad Nacional Autónoma de México, Cuautitlán Izcalli, Estado de Mexico C.P. 54740, Mexico; amirakhalil2001@yahoo.com.mx (A.J.F.-M.); renegerardo.escobedo@gmail.com (R.G.E.-G.); garroyo@unam.mx (G.A.A.-R.); olinoco@yahoo.com.mx (M.O.N.-C.); mirruv@yahoo.com.mx (R.M.-R.)

**Keywords:** boronic acids, diindolylmethane, spectroscopy, DFT calculations, hydrogen bonds

## Abstract

The structure of the *ortho*-, *meta*- and *para*- hybrid diindolylmethane-phenylboronic acids and their interactions were optimized with by a quantum chemical method, using density functional theory at the (DFT) level. Thus, infrared bands were assigned based on the scaled theoretical wavenumbers by correlating the respective experimental data of the molecules. In addition, the corresponding ^1^H-/^13^C-/^11^B-NMR experimental and theoretical chemical shifts were correlated. The target molecules showed a poor treatment of the OH shifts in the GIAO method due to the absence of explicit solvent effects in these calculations; therefore, they were explicitly considered with acetone molecules. Moreover, the electron density at the hydrogen bond critical point increased, generating stabilization energy, from weak to moderate or weak to strong, serving as an indicator of the strength of the hydrogen bond between the different intermolecular interactions. Finally, some properties related to the reactive behavior of the target molecules associated with their cytotoxic effects and metabolic pathways were also calculated.

## 1. Introduction

Boron-containing compounds are substances with a wide range of potential applications in the fields of material science, supramolecular chemistry, catalysis and biology. In recent years, this class of molecules has become an object of increasing interest due to the bioactive nature of some of them [1]. In particular, the case of boronic acids should be particularly highlighted, since some of have insecticidal, fungicidal, antibacterial and antineoplastic activities, e.g., bortezomib (Velcade^®^) [2]. Boron−based compounds also show preferential localization in tumor tissues, thus making possible the so called boron-10 neutron capture therapy [3,4]. In addition, the boronic acid moiety has also been used in the synthesis of antitumor and antiviral agents [3,5].

Phenylboronic acids are boronic acid derivatives with some of the previously mentioned biomedical applications, such as diphenylboronic esters with antibacterial activity, or phenylboronic acid (PBA) and diphenylboronic esters (DPBE) which are the most efficient types of boronic acid derivatives that act as serine protease inhibitors decreasing cancer cell viability, being in this application PBA more efficient than boronic acid as the cancer cell viability decreases within eight days [6,7].

On the other hand, 3,3′-dindolylmethane (DIM) is an important organic compound derived from the acid catalyzed condensation of a natural product, indol-3-yl-carbinol (IC3), a molecule produced as an artifact from cruciferous vegetables [8]. I3C is an anti-tumor agent and several studies have revealed its clinical efficacy against various epithelial cancers, including endometrial and mammary tumors [9]. Moreover, DIM and some of its analogues (DIMs) are currently among the most popular adjunct therapies for recurrent respiratory papillomatosis due to their effectiveness and low level of toxicity [10,11].

It is also convenient to highlight that modern synthetic chemists currently engage in the synthesis of hybrid molecules, which are made by combining appropriately pieces of different other molecules, in order to synergize or modify the pharmacological activity of the corresponding prototypes. As part of our ongoing research program, we are interested in green chemistry protocols for the production of novel hybrid heterocyclic molecules, and thus we recently reported the synthesis and biological evaluation of three relatively novel hybrid molecules **I**–**III** of diindolylmethane and phenylboronic acids [12]; it is important to note that the obtained results were indicative that the hybrid products were promising antineoplastic compounds. To this end the cytotoxic effects of the hybrid molecules were evaluated on six cancer cell lines (U251 = central nervous system glia, PC-3 = prostate, K562 = leukemia, HCT-15 = colon, MCF-7= breast, SKLU-1 = lung), which evidenced that the *meta* regioisomer was the most active [12]. Consequently, taking into account the importance of these compounds, we deemed it convenient to report the novel and interesting results of a theoretical study of the target compounds **I**–**III**. Using quantum chemistry biological activity predictor methods we determined: (i) the respective conformational analysis; (ii) the fully optimized molecule structures, determining the geometrical parameters: including bond lengths and angles; (iii) also the infrared spectrophotometric data and NMR (^1^H-, ^13^C-, ^11^B-) chemical shifts, were determined in the gas-phase and also considering acetone as solvent. Furthermore, (iv) molecular predictions regarding the human metabolism of the target compounds using metaprint 2D were also evaluated.

## 2. Results and Discussion

### 2.1. Molecular Parameters

#### 2.1.1. Energy

##### Conformational Analysis

**Figure 1:**
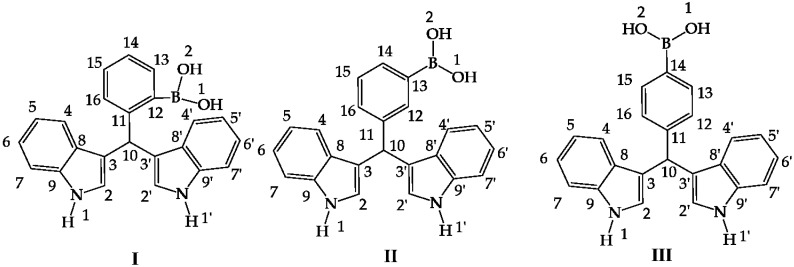
Hybrid diindolylmethane phenylboronic acid regioisomers: *ortho* (**I**), *meta* (**II**) and *para* (**III**).

In order to calculate the conformers of each regioisomer **I**–**III** of the target diindolylmethane phenylboronic acids (Figure 1), they were in a first instance optimized by B3LYP with 6311++G(d,p).

**Figure 2:**
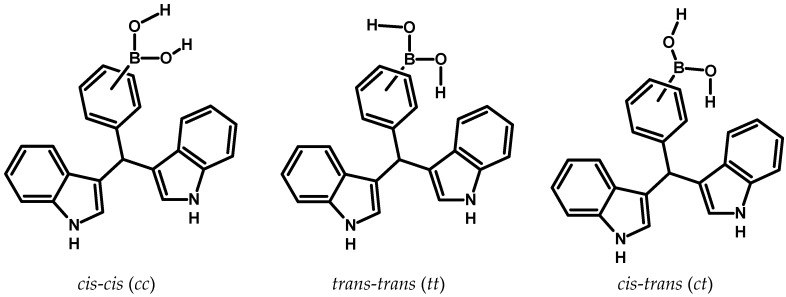
There is the possibility of forming three possible conformers, this depends on the location of the hydrogen atoms that are bonded to the oxygen atoms of the –B(OH)_2_ group.

It is convenient to note that the boronic compounds have three possible conformers depending to the location of the hydrogen atoms bonded to the oxygen atoms in the –B(OH)_2_ group; consequently, they can be oriented toward the benzene ring (*trans*-*trans* = *tt*), opposite to benzene ring (*cis*-*cis* = *cc*) or alternated (*cis*-*trans* = *ct*) (Figure 2).

The conformer energy values of the studied compounds are listed in Table 1. The comparison of the values indicates a slight energy difference (0.7 to 3.3 kcal/mol), and it can also be seen the the *cis*-*trans* configuration resulted as the most stable among the three regioisomers. These results agree with previously reported works for other boronic acids [1,3,13,14,15]. Among the three possible *cis-trans* isomers, the *ct*-**III** appears to be the most stable, followed by the *ct*-**II** and *ct*-**I** ones. The energy value difference between the less and more stable ones is 3.1 kcal/mol. The variations in the relative energies among **I**–**III** are small, suggesting that these isomers are almost equally stable. The stability of *ct*-**III** molecule can be attributed to the lower steric effect. These stability results have been previously reported [12].

For the optimized structures, the hydrogens atoms were in the O–B–O plane. Most probably, this is due to oxygen lone pair electrons being in resonance with the *p* orbital empty of the boron, forcing the hydrogens atoms to be in the same plane. In all the conformers of **I**–**III**, the boronic acid group was planar and remained in the same plane as the benzene ring or close to it.

##### C–B Bond Rotational Barrier Analysis

It is worth nothing that the effect of the aromatic ring and conformational arrangement in the rotational barrier of the C–B bond was also studied at the same theory level [3], considering a C12–C13–B–O dihedral angle of 90° (Table 2). The analyzed conformers for the C–B bond show that the oxygen of the boronic group is in a perpendicular plane, in comparison to the previously evaluated conformer (Table 1). These structures **I**–**III** showed imaginary frequencies in all cases, demonstrating that all of them correspond to transition states or unstable structures, and in this sense the difference with the coplanar structure was the energetic barrier for the bond rotation. The energy values of each structure with dihedral angles of 90°, the energies of the coplanar structures and their difference are listed in Table 2. The comparison between the 90° conformers does not show great energy difference values (between 1.3–5.0 kcal/mol).

The *cis*-*trans* and *cis*-*cis* conformers were calculated when the hydrogen atoms were bonded to the oxygen atoms in the –B(OH)_2_ group are in the plane, and when they are perpendicular (90°). As it can be seen in Table 3, an increasing trend in the rotational barrier is observed, with an increase of the distance between the indolylic and the boronic moiety. The conformers (*cis*-*trans* and *cis*-*cis*) of **I** and **III** displayed the same trend. For ΔE_**I–I**−90°_, their energy values were 1.0 and 2.7 kcal/mol, respectively, as a consequence of the steric effect due to the proximity of the diindolylmethane moiety. On the other hand, greater rotational barrier values (2.7 and 5.9 kcal/mol, respectively) were obtained for ΔE_**III–III**−90°_, which is associated to an increase in the C→B π bonding, due to the presence of the diindolylmethyl group in the C11 position [16].

The analysis of the *trans-trans* conformers, calculated when the hydrogen atoms bonded to the oxygen atoms in the –B(OH)_2_ group are in the plane and when they are perpendicular (90°), revealed a different behavior. The isomer **I** possess the greater energy barrier as a consequence of an interaction of the hydrogen atom of the boronic group with an indolyl group in the conformer without torsional effect. This interaction was evaluated by the AIM method, developed by Bader and co-workers, in the analysis of bond critical points of electron density (BCP’s), and the gradient paths for the structure [17,18], was discussed in the next section.

**Figure 3:**
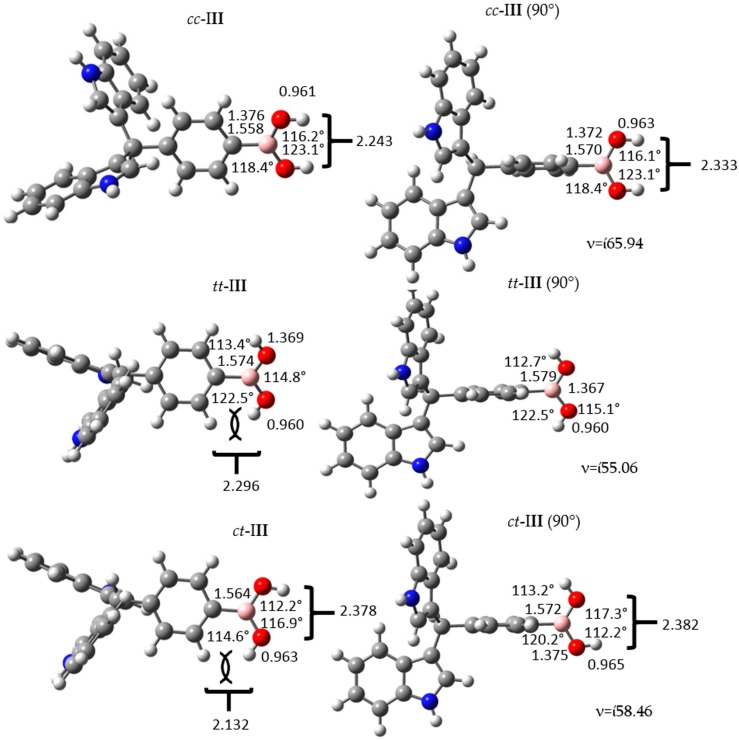
Structure and geometrical parameters of the *p*-diindolylmethane phenylboronic acid hybrid isomers and their corresponding 90° conformers. The gray, blue, pink, red and white spheres are for carbon, nitrogen, boron, oxygen and hydrogen atoms respectively.

The conformational structures of the *p*-regioisomer (maximum stability, **III**) at 90° are displayed in Figure 3. All conformers with 90° have imaginary frequencies. Additionally, some geometrical parameters of the boronic acid group changed.

The σ bond distance among C–B increased in all 90° conformers, as a consequence of scission of conjugation between the aromatic ring and the unoccupied *p* orbital of the boron atom, present in the boronic acid group. In addition, the difference in C–B bond lengthen conformers *tt*-**III** and *ct*-**III** was lower (0.009 Å), which may be due to the weak interaction between the electronic pi cloud of the benzyl ring, and the hydrogen atoms of the boronic group. Furthermore, the O–H and O–O bond lengths in the boronic acid group increased, while the B–O distance decreased. This behavior, was constant in all the studied isomers.

#### 2.1.2. Geometrical Parameters

##### Geometry of **I**–**III**

The bond lengths of the conformers of the studied molecules were determined at the same level of theory (Table 4). The theoretically calculated data did not show great differences in the length, between the conformers; the most significant change in the bond lengths was observed in the boronic groups by the steric or electronic effects associated with their arrangements. Regarding the *ct* and *tt* conformers of **I**, the previously indicated effect of the intramolecular interaction was observed in the C–B distance, which was decreased by the attractive force of C3′.

Since the experimental X-rays patterns of the target molecules have not been yet published, and considering the motifs present in these molecules [1,3,12,14,19,21,22], the obtained values were compared by the same motif previously reported in other compounds [14,19,20]. In this sense, the calculated values were agreed with the previously reported data, considering the structural differences with the reference molecules. Taking into account the most stable conformer (*ct*), the C–B bond distance in the three target molecules was lower than the reference (pentafluorophenylboronic acid) [14], this difference was attributed to the absence of the fluorine atoms in the latter structure [14,22]. Furthermore, the comparison of the same conformer (*ct*) of the three analyzed compounds, the distance C–B in **I** is greater than **II** and **III;** this increase was considered a result of the steric stress between the boronic acid group and the indolylic moiety.

Additionally, the distance of the bond B–O increased in the target molecules, in comparison with the reference, indicating less delocalization of the unshared electron pair of the oxygen to the boron atom as a consequence of the lack of fluorine atoms in the aromatic ring.

Following the geometrical parameters, the calculated bond angles (Table 5) in the considered conformers were contrasted. These parameters, as expected, were very similar in the phenyl and indolylic moieties [20], the biggest changes being observed for the boronic acid group. Related to the boronic acid group angles, the *tt* conformers showed higher values in regard to other conformers, except in the case of the C–B–O motif, which presented in all cases the smallest angle value. The theoretical angle was compared with the same references, observing that they are in agreement with the corresponding experimental values. Thus, it is important to note, that the best approach of the angle values corresponded to **I** in its *ct* conformer, according to the experimental values.

The molecule **III**, the most stable under consideration, was characterized by the presence of intermolecular interactions, which can be decisive in the crystal packing of this kind of products. It is worth noting that in previous works the structural crystal description of some phenylboronic acid derivatives has been reported. According, to a low-temperature study, for the unsubstituted phenylboronic acid crystals [22], they were formed by an asymmetric unit, composed by two molecules of the acid, achieving a dimer. The study with **III** was made assessing that a dimer structure and the non-covalent interaction could be present. The proposed interactions will be discussed later in the paper.

##### Intramolecular Interaction for Molecule **I**

**Figure 4:**
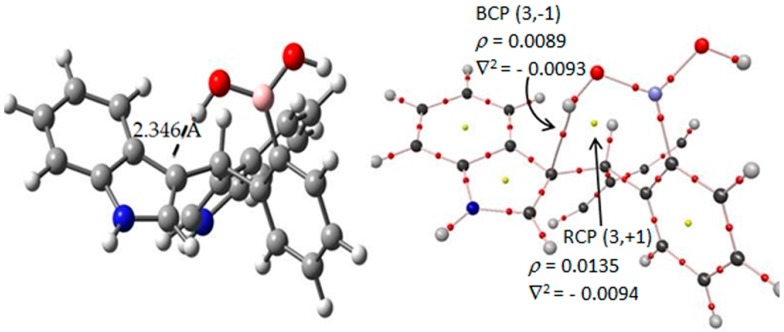
AIM results of the hydrogen–C3′ interaction in the *tt*-**I** diindolylmethane-phenylboronic acid hybrid. Molecular graphs for *tt*-*ortho*-regioisomer interaction. Density *ρ*(*r*) and its Laplacian ∇^2^*ρ(r)* at the BCPs characterizing the HB interactions. The gray, blue, pink, red and white spheres are for carbon, nitrogen, boron, oxygen and hydrogen atoms respectively.

As previously described, the intramolecular interaction present in the *tt*-**I** conformer was analyzed by the AIM method. These results are displayed in Figure 4, the molecular graph shows the bond critical points of electron density (BCP’s) (3,−1) located at OH●●●CH, and this contact suggests the existence of a chemical interaction. In fact, the density *ρ*(*r*) was 0.0089 e/a.u.^3^ and the Laplacian ∇^2^*ρ*(*r*) was −0.0093 e/a.u.^5^ meanwhile the ring critical point (RCP) (3,+1) had a density value (*ρ*(*r*)) of 0.0135 e/a.u.^3^ and the ∇^2^*ρ*(*r*) was −0.0094 e/a.u.^5^. The negative Laplacian values indicated a local concentration of charge, suggesting an interaction of the lone electron pair type or Lewis base, as a model of reactivity [21]. This behavior can be explained by means of a resonance effect of the non-shared electron pair of the nitrogen atom within the indole ring, causing the presence of an electronic pair in the C-3’ position of the indole ring and the attraction of the hydrogen atom.

As a consequence of this interaction a large rotational energy barrier was achieved. This is in addition to the lower energy difference values between the *cis*-*trans* and *trans*-*trans* conformers of **I** (Table 1) resulting in the stabilization effect of this interaction. Regarding **II** and **III** in the *trans*-*trans* conformers the energy barriers provided the same behavior, previously mentioned, however the *tt*-conformers have less energy difference than the *cc* and *ct* conformers. This decrease was attributed to a steric repulsion between the hydrogen of the boronic acid group and the hydrogen at C-13 (–OH●●●H–C13 length around 2.2 Å) by the hydrogen orientation; this repulsion 0.indicated that the boronic acid groups do not stay in the same plane of the benzene ring.

##### Intermolecular Interactions for Molecule **III**-2

**Figure 5:**
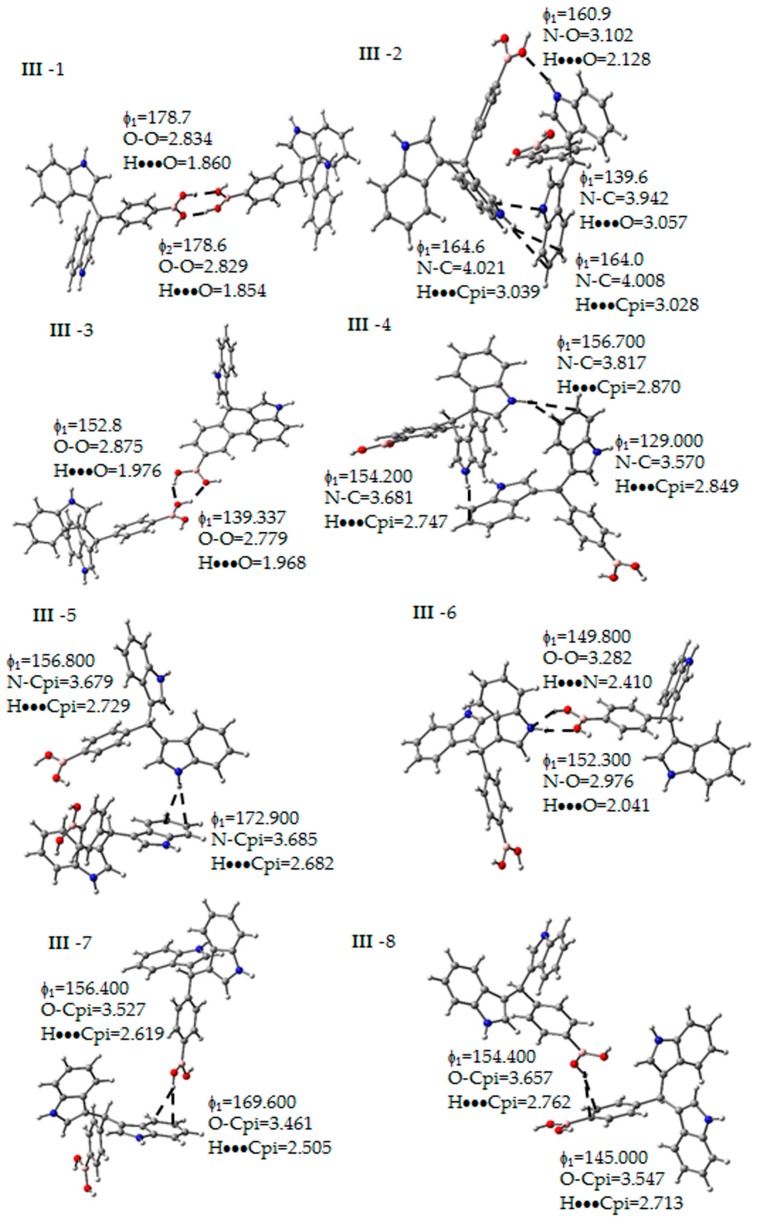
Non-covalent interactions determined between the molecules of **III**. Some geometrical parameters as bond length and angle are next to the bond. The gray, blue, pink, red and white spheres are for carbon, nitrogen, boron, oxygen and hydrogen atoms respectively.

The presence of eight dimeric structures was observed. In this sense, two principal intermolecular interactions were found: (a) hydrogen bonds between the hydrogen atoms of the boronic acid group (**III**-1 and **III**-3, Figure 5) or the N–H bond of the indolylic moiety with the same groups, the boronic acid groups (**III**-2 and **III**-6, Figure 5), and (b) the hydrogen of the boronic acid or N–H bond of the indolyl group, with the π-aromatic systems (**III**-2, **III**-4, **III**-5, **III**-7 and **III**-8, Figure 5). The corresponding electronic and interaction energies values for the dimers are summarized in Table 5. The first dimer analyzed was **III**-1, which according to Figure 5, presented two intermolecular hydrogen bonds among the boronic acid groups; a smaller distance between the acceptors and donors (approximately 1.86 Å) and an angle closer to 178.7° that indicated a strong intermolecular interaction. Additionally, the electronic energy and the interaction energy showed bigger values in contrast with the other dimers, confirming the stronger character of these hydrogen bonds. This type of dimer has been previously reported for the crystalline structure of phenylboronic acid [22] and these theoretical values agree with the experimental data reported for phenylboronic acid (distances: hydrogen-acceptor 1.81 Å, between donor and oxygen acceptor 2.72 Å and bond angle 175°).

Additionally, there is a different kind of hydrogen bonds between the boronic acid groups, as can be seen by comparing **III**-3 and **III**-1. The **III**-3 dimer revealed the presence of a bifurcate acceptor in the hydrogen bond [23], decreasing the stability of the interaction, in comparison with **III**-1, due to the increase (1.97 Å) of the bond length and the decrease of the angle value (130°–150°). Finally, **III**-6 showed hydrogen bonds between a boronic acid group and the N–H of the indole motif, and the interaction properties (Table 6 and Figure 5) indicated a weaker bond [23], distance of 2–2.4 Å, ф around 150°) and the interaction energy exhibited the lower value (−2.5 kcal/mol) than the dimers with only hydrogen bonds.

Regarding the dimers **III**-2, **III**-4 and **III**-5, they presented an interaction between hydrogen atoms and the π-aromatic systems of the indoles; this type of interaction has been previously described [20,24]. In the references, the crystal packaging of the molecules is stabilized by different weak intermolecular contacts such as the hydrogen bonds and they were formed by means of folded layers. Moreover, the cohesion between these layers results from the C–H●●●π intermolecular interactions, and analogous N–H●●●π contacts, were observed for 3,3′-diindolylmethane derivatives [20,24]. Concerning the calculated bond parameters, the distances between the hydrogen and the π-system are 2.500 to 3.000 Å; additionally, the bond angles had values between 150°–170°, which are in agreement with the values obtained for 3,3′-diindolylmethane derivatives that had a bond distance of 2.750 and 2.470 Å, and the bond angle are in the range of 148° and 171°. According to the second kind of the interaction, N–H●●●π, the most stable of this group of dimers was **III**-2, which additionally, has a hydrogen bond among the boronic acid and indolylic moiety, while the **III**-7 dimer presented an interaction between the hydrogen atoms of the boronic acid group and the π-aromatic system of the indole and the dimer **III**−8 indicating an interaction between a hydrogen atom of the boronic acid group and the π-aromatic system near the boronic acid group.

The dimer **III**-4 presented two N–H●●●π interactions, with bond distances among the hydrogen and π system in the range of 2.7–2.8 Å, acceptor and donor between 3.6−3.8 Å and bond angles of 150° and 152°. This corresponds to weak hydrogen bonds [23]. Finally, **III**-5 has only one interaction of this type; it was the most unstable dimer of the group.

**Figure 6:**
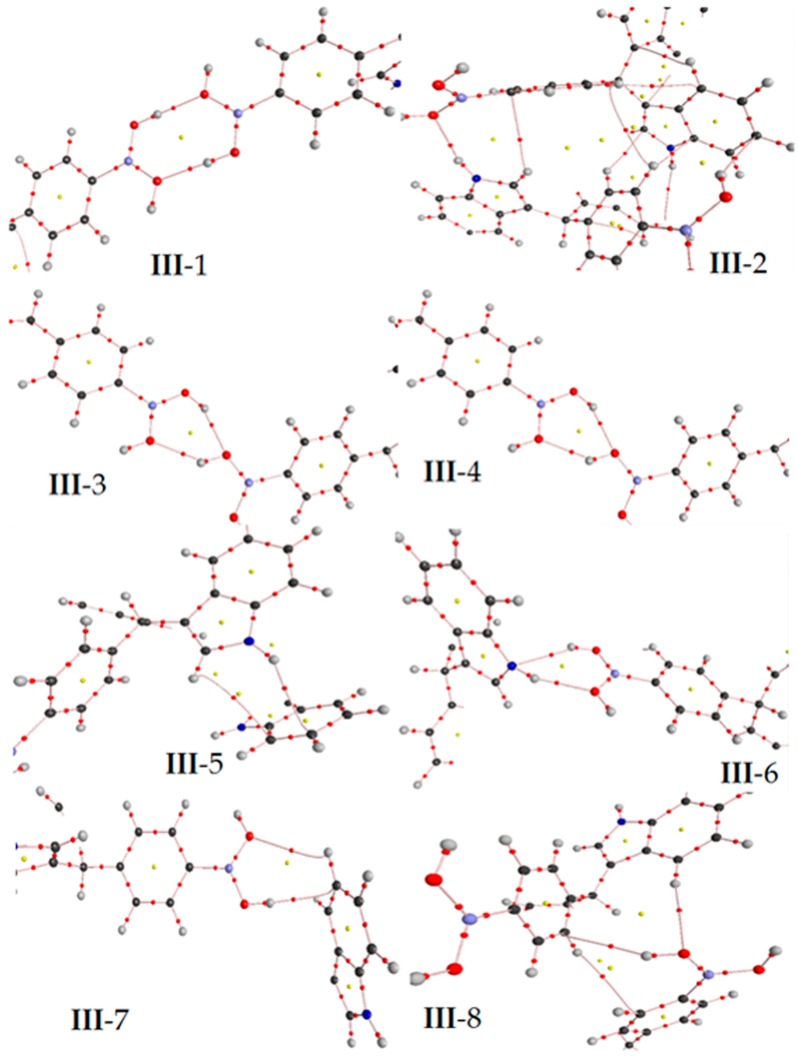
In the target molecule (*ct*-**III**) the bond critical points (3,−1) is marked in red small points and ring critical points (3,+1) are presented as yellow points. The gray, blue, pink, red and white spheres are for carbon, nitrogen, boron, oxygen and hydrogen atoms respectively.

Complementarily, considering the dimers, and using the atoms in molecules methodology to find the critical points of electron density in order to obtain the parameters *ρ*(*r*) and the Laplacian ∇^2^*ρ*(*r*), were studied. The corresponding values are summarized in Table 7; also, Figure 6 shows the structure with the critical points (3,−1) and (3,+1). These results confirmed the presence of the interactions, having positive density values, with respect to the OH●●●OH and NH●●●OH interactions, the values were in the order of 10^−2^ e/a.u.^3^, typical data of the range of hydrogen bond [25]. In this sense, the density values of the hydrogen atom●●●π system and the critical points of rings showed values in the order of 10^−3^ e/a.u.^3^ corresponding to Van der Walls complexes [26]. Thus, all of the Laplacian values were negative, indicating a local charge concentration [21], assigned to a shared interaction, corresponding to the typical valence shell charge concentration [21,25,26].

#### 2.1.3. Spectroscopic Properties

The theoretical calculations for the vibrational modes in infrared spectroscopy and the chemical shifts in nuclear magnetic resonance were performed for the most stable conformer of the studied molecules (*cis*-*trans*). The obtained data were compared with the corresponding experimental values in order to make a detailed analysis of both the vibrational modes and the respective shielding or deshielding phenomena.

##### Infrared Spectra

**Figure 7:**
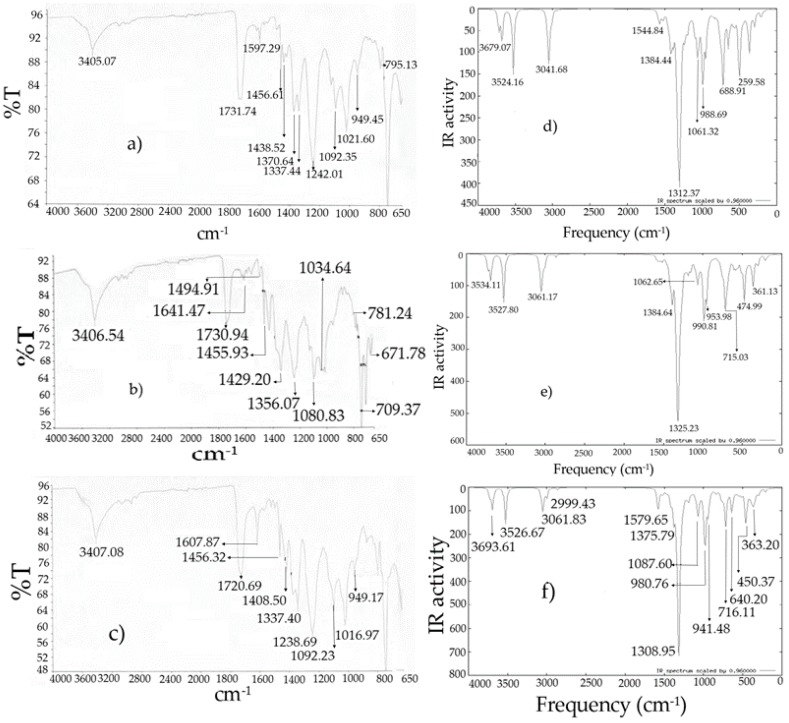
Infrared spectra: (**a**) **I** experimental [10]; (**b**) **II** experimental [10]; (**c**) **III** experimental [10]; (**d**) **I** theoretical; (**e**) **II** theoretical; (**f**) **III** theoretical.

The infrared spectra calculations were made for a free molecule in the gas phase, while the experiments were performed for solid samples considering the use of KBr to prepare a pellet. Each target molecule **I**–**III** consists of 47 atoms, with 135 normal vibrational modes. These normal vibrations are distributed 91 in-plane and 44 out of plane. All the vibrations were active in the IR absorption spectrum. The selected wavenumbers data of the molecules of infrared theoretical and experimental were summarized in Table 8; additionally, the theoretical and experimental IR spectra are displayed in Figure 7.

The assignments of the vibrational absorptions were made by comparison between related molecules reported in the literature and those results obtained from the respective theoretical calculations [1,3,14,15]. The descriptions of the presented modes were approximated, being convenient to note that some of the vibration were mixed together. Regarding the results, the presence of four bands in the range of 3700–3500 cm^−1^ were observed for the target molecules (Table 8 and Figure 7), where the two bands at high frequencies correspond to the OH stretching of the boronic acid group [1,3,14,15,22] and the next bands were attributed to N–H stretching of the corresponding indolylic moieties. It is convenient to note, that in the experimental spectra a broad band around 3400 cm^−1^, was assigned to the mix of the aforementioned bands.

The C–H stretching frequencies appeared in the range of 3100–3000 cm^−1^ corresponding to an aromatic compound and in the range of 2850–3000 cm^−1^, for an aliphatic molecule. In this sense, the symmetrical stretching of the aromatic indolylic and benzene rings were observed in the range 3060–3050 cm^−1^, the bands from 3050 to 3040 cm^−1^ of C–H asymmetrical stretching of the indolyl group, and of the benzene ring hydrogens. In the experimental spectra the symmetrical and asymmetrical stretching bands were observed in the ranges of 3100–3000, 3000–2900 and 2900–2800 cm^−1^, respectively. The theoretically scaled frequency and the experimental data were in agreement [27,28].

The C=C stretching and bending in aromatic compounds are usually found between 1620–1680 cm^−1^ and 1500–1700 cm^−1^, respectively. In the target compounds, intense bands around 1700 cm^−1^ were obtained in the experimental procedure and in the computed values these were perceived in the range of 1630–1580 cm^−1^; these correspond to the symmetrical stretching of the indolylic moiety and the benzene ring [27,28]. An experimental band around 1600 cm^−1^ was assigned to the asymmetrical stretch of the indole and the benzene ring, in addition to the bending of C–H and N–H bonds, that were correlated with the theoretical values at 1580–1490 cm^−1^.

The B–O asymmetric stretching band of phenylboronic acid occurs around 1390–1330 cm^−1^ [14,21,29], and the corresponding experimental infrared spectrum showed a band at 1336–1337 for the diindolylmethane phenylboronic acid hybrids. The calculated band appeared in the range of 1360–1330 cm^−1^, in other words, the theoretical and experimental values are in accordance. The B–C stretching band according to the literature is found in the range of 1080 and 1110 cm^−1^ or up to 1300 cm^−1^ [3,14,15,27,28,29]. For the studied molecules, the experimental bands were found close to 1090 cm^−1^ meanwhile the calculated values were around 1100–1070 cm^−1^. Finally, the B–OH bending band was observed in the range of 1039–1017 cm^−1^ while in the target molecules it was in the theoretical range of 1030 and 960 cm^−1^. In general, the mentioned values agree.

##### NMR Spectroscopy

**Figure 8:**
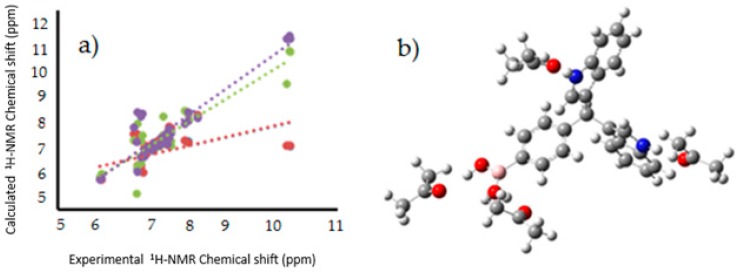
(**a**) Linear regression between experimental [12],and calculated ^1^H-NMR chemical shifts for the target molecules in PCM (Red), explicit interaction (Green), PCM and explicit interaction (purple); (**b**) Model of explicit interaction for **III** with four acetone molecules. The gray, blue, pink, red and white spheres are for carbon, nitrogen, boron, oxygen and hydrogen atoms respectively.

An interesting outcome was obtained from the corresponding theoretical study of the Nuclear Magnetic Resonance (NMR) chemical shifts for ^1^H, ^13^C and ^11^B, both in gas phase and acetone solution. It should be mentioned that the corresponding experimental values of the chemical shifts for ^1^H and ^13^C have been previously reported by our research group [12], and these values were correlated with those calculated by DFT at the B3LYP level using the 6-311++G(d,p) basis set and the GIAO method (Figure 8).

Thus, the GIAO methodology and Tomasi’s polarizable continuum model (PCM) were used, considering the agreement among experimental and theoretical data observed in previous works [30,31]. However, in this case, the linear regression analysis of the data set of ^1^H-NMR shifts with the gas phase data, provided a regression coefficient of 0.1047, indicating a disagreement in the theoretical and experimental data. The review of the calculated values showed greater variations in the hydrogens of the boronic acid group and in the nitrogen in the indolyl groups. These differences were attributed to the hydrogens located on the periphery of the molecule, more susceptible to intermolecular interactions, resulting in a deviation from the theoretical values.

The computational study was carried out using the solvent effect of acetone in explicit and implicit modes. In a first approach, the solvent effect was studied using the self-consistent reaction field (SCRF) method and considering Tomasi’s polarizable continuum model (PCM) at the same theory level aforementioned. The chemical shift results with this procedure also disagreed with the obtained experimental results in the linear regression analysis, providing a regression coefficient of 0.105, which is slightly different from the gas phase value, overlapping the line with the gas phase line. In addition, explicit interactions between the solvent, in this case acetone, with the hydrogen of the boronic acid group and the nitrogen atom of the indolyl groups, were established and their chemical shifts consequently calculated. The results were similar to the experimental values, giving a regression coefficient of 0.8224, confirming the explicit interactions with the solvent. Finally, the chemical shifts were also determined using the mix of PCM and explicit interaction obtaining a regression coefficient of 0.8322, that means a slightly increase in the experimental value approximation. The equation used to describe the last fit was δH_theo_ = 1.159δH_exp_ − 0.726 ppm, where δH_theo_ is a chemical shift predicted on the basis of the experimental values δH_exp_. The slope and intercept showed a standard deviation of 0.089 and 0.660, respectively.

**Figure 9:**
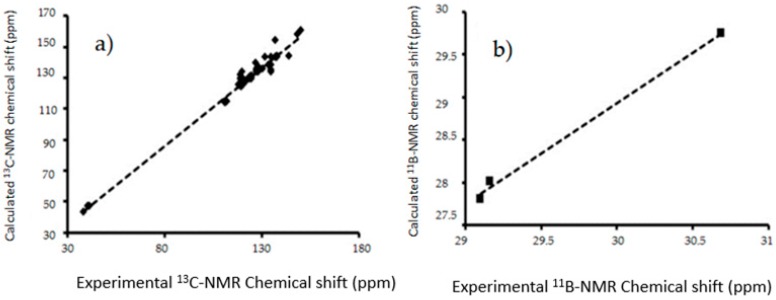
The linear regressions between experimental and B3LYP/6-311++G(d,p) calculated (**a**) ^13^C-NMR chemical shifts for in gas phase (**b**) ^11^B-NMR chemical shifts in gas phase **I**–**III**.

A similar analysis was made for the theoretical and experimental chemical shifts obtained for ^13^C- and ^11^B-NMR, and the corresponding scheme is shown in Figure 9. In contrast with the ^1^H-NMR calculations, the gas phase computed chemical shifts for ^13^C and ^11^B agreed with the experimental values, with regression coefficients of 0.979 and 0.996, respectively. This concordance, in contrast with the ^1^H predictions is a consequence of the lesser susceptibility of the boron and carbon atoms to intermolecular interactions in comparison with the hydrogen ones.

The equations to describe the fit of ^13^C and ^11^B were δC_theo_ = 1.0148δC_exp_ + 4.7488 ppm, with a slope and intercept standard deviation of 0.0187 and 2.3004, respectively and in the case of ^11^B, δB_theo_ = 1.1803δB_exp_ − 5.8856 ppm, and a slope and intercept standard deviation of 0.0747 and 2.1786, respectively. In other words, the values of the regression coefficients reflected a good description of the experimental chemical shifts by the selected method, both at a theory and the basis set level.

### 2.2. Reactivity Parameters

The reactivity parameters of the title molecules—hardness (η), chemical potential (μ) and electrophilicity (ω)—were calculated considering the electronic affinity (*EA*) values and their ionization energy (*I*). The cation and anion energies of the studied molecules were calculated at the same level and were used for the *EA* and *I*. The obtained results as well as the corresponding previously evaluated biological activity [12] are summarized in Table 9. Taking into account the respective values of the electronic affinity, **I** showed the greater value in comparison with the minor value of **III**. This behavior was explained considering the stability of **III** in its neutral form; furthermore, this result could be related with the corresponding lesser biological activity. On the other hand, the ionization energy, the hardness, in addition to the chemical potential, nucleophilicity and electrophilicity followed a behavior from major to minor value in the order of **III** > **II** > **I**. This outcome was contrasted with the percentage of growth inhibition in some cell lines and some trends were found. For example, **III** showed the bigger hardness values agreeing with the lesser reactivity, while marked and lower activity was observed in all the cell lines.

It is convenient to note that the high reactivity of **I** could cause an interaction with some molecules present in the cytoplasm, e.g., sugars, and not reactions with the target receptors or proteins [6,32,33], for the cytotoxic effect. In addition, the activities of **I**–**III** in the PC-3 cell line were inversely proportional to their reactivity values, being **I** the most active.

Finally, the activity in the rest of the cells lines followed the trend of the cationic species stability in which **II** showed the higher activity. It is important to note that this correlation is a worthy subject of study for subsequent works using biological tests and in silico studies.

### 2.3. Metabolic Products as Bioactive Molecules

**Figure 10:**
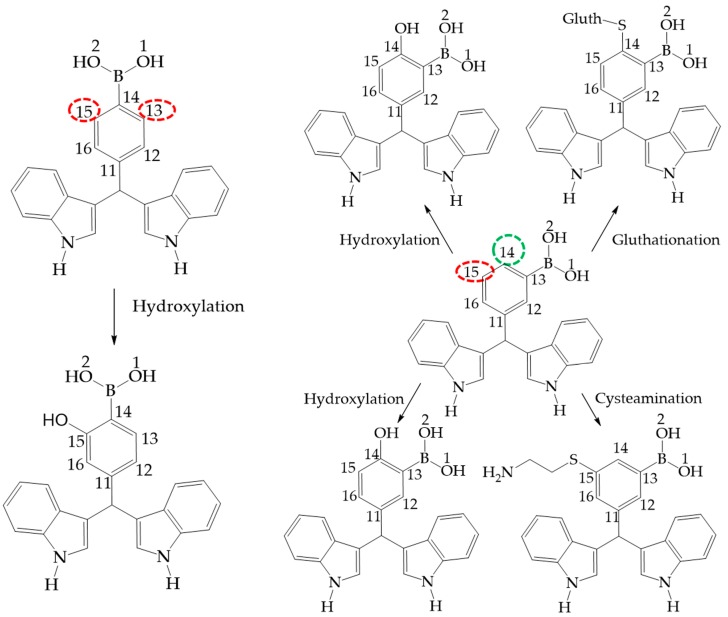
Phase 1 metabolic pathways of **III** and **II**. The red color indicates high values of NOR, from 0.66 to 1. The green color indicates a NOR value range of 0.15 to 0.33.

**Figure 11:**
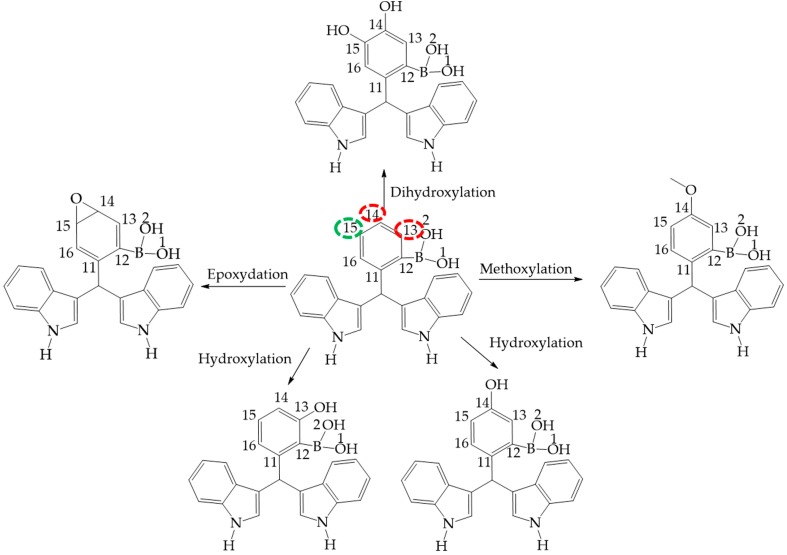
Phase 1 metabolic pathway of **I**. The red color indicates high values of NOR, from 0.66 to 1. The green color indicates a NOR value range of 0.15 to 0.33.

It is appropriate to highlight the fact that the target molecules are promising compounds with possible uses as drugs [12]. This study establishes plausible degradation pathways for the phase 1 and 2 human metabolism, that can be predicted using the program metaprind2D [35,36,37]. The corresponding results are displayed in Figure 10 for **III** and **II**, while the suggested metabolism of **I** is shown in Figure 11. It is also convenient to note that **I** and **III** correspond to the more and less reactive molecules, respectively, a status reflected in the number of metabolites predicted. In Figure 10 and Figure 11, the corresponding colors used to highlight atoms indicate the normalized occurrence ratio (NOR).

A high NOR indicates a more frequently reported site or metabolism in the metabolite database. The red color indicates high values of NOR, from 0.66 to 1; the orange color designates values between 0.33 to 0.66, the green color indicates a NOR value range of 0.15 to 0.33, the white color, range values of 0.00 to 0.15 and finally grey denotes lack of data. It is important to highlight that in the studied molecules only red and green colors were found. Moreover, the number of metabolites predicted was correlated with the local reactivity parameters as the Fukui (*f*^−^) and Parr indexes, the local nucleophilicity and electrophilicity (P^−^, P^+^, *N*_k_, ω_k_) [34,38], which are displayed in Table 10.

It is noted that the reactive centers indicated by the metaprind2D program correspond to the atoms in the phenylboronic acid moiety with the less steric effect and with better values of local reactivity. In case of **II**, the biggest value of electrophilicity and less steric hindrance is found at C14 (ω_k_ = 0.090) being this an electrophilic center; in this sense C15 has a big value of nucleophilicity (*N*_k_ = 0.210); in that sense C15 in **III** had values of 0.030 corresponds to the most electrophilic center with less steric effect (*N*_k_ = 0.030).

The predicted metabolites correspond to the reaction of **III** and **II** with reactive oxygen species (ROS) indicating antioxidant character [6,39,40]; Complementarily, Figure 11 shows the metabolism products of **I**. The reactive sites also agree with the most deactivated nucleophilic centers (C14 = −0.21 and C15 = −0.280) and the best electrophilic centers without steric hindrance (C14 = 0.040 and C15 = 0.130) showing the same behavior in the hydroxylated derivatives production. However, these results predicted the possibility of elimination by the action of glutathione *S*-transferase, a detoxification enzyme [41], in addition to the reaction of **II** with cysteamine.

## 3. Methods

### 3.1. Calculational Methods

#### 3.1.1. Density Functional Theory

The molecular mechanics search for conformers of the diindolylmethane phenylboronic acids (**I**: *ortho*, **II**: *meta*, **III**: *para*) and their intermolecular interactions [13], were determined using the PC Spartan’06 program [42]. The corresponding molecular geometry and all the calculations were performed using the Gaussian 09 program [43]. The Becke’s three-parameter hybrid density functional, B3LYP [44,45], was used to calculate geometry optimization, electronic and spectroscopic properties with the 6-311++G(d,p) basis set [46,47,48,49]. The optimization was carried out using the Berny analytical gradient optimization methods [50,51,52].The first step of the calculations also yielded the distribution of atomic net charges.

#### 3.1.2. Atoms in Molecules

The intra and intermolecular H bonding (HB) interactions were analyzed using Bader’s topological analyses based on atoms in molecules (AIM) theory [17,18,53]. This type of analysis provides critical points of *ρ*(*r*) and it’s Laplacian, ∇^2^*ρ*(*r*). The critical points of *ρ*(*r*), which present two negative curvatures and one positive curvature, identify the bonds in the molecule and will be denoted hereafter as bond critical points (BCPs). The critical points with one negative and two positive curvatures are associated with the existence of a ring structure and will be denoted as ring critical points (RCPs). The values of *ρ*(*r*) and ∇^2^ *ρ*(*r*) at these points provide quantitative information on the strength and nature of the bonding and the characteristics of the ring. The Laplacian of the electronic charge density, identifies regions of space where *ρ*(*r*) is locally concentrated, ∇^2^*ρ*(*r*) > 0, or depleted, ∇^2^*ρ*(*r*) < 0. In general, negative values of ∇^2^*ρ*(*r*) are typical of covalent interactions, whereas interactions between closed−shell systems are characterized by positive values of ∇^2^*ρ*(*r*). Some topological criteria of the existence of HB have been proposed [54,55,56]. The following are the most common criteria applied to study HB interactions:

- A BCP must be located within the H●●●A contact region, which topologically proves an increase in the density and consequence the existence of HB;
- The value of the electron density of the BCP arising from the H●●●A interaction, which should range between 0.002 and 0.040 a.u.;
- The value of the corresponding Laplacian, which should range from −0.139 to −0.024 a.u.;

The AIM analyses were performed with the AIM2000 computer algorithm [57].

#### 3.1.3. Vibrational Analysis

Density functional theory calculations are reported to provide excellent vibrational frequencies of organic compounds, if the calculated frequencies are scaled to compensate for the approximate treatment of electronic correlation, for basis set deficiencies and for the anharmonicity [58,59,60,61].

Rauhut and Pulay [58] calculated the vibrational spectra of thirty-one molecules by using B3LYP methods with 6-31G(d) basis set, which can be used in order to eliminate the uncertainties in the fundamental assignments in infrared spectra. Consequently in this work, by using the DFT (B3LYP/6-311++G(d,p) method, the respective vibrational frequencies were calculated, and the molecular energy was differentiated twice with respect to the Cartesian coordinates of the diindolylmethane atoms in the ground state. The outcome represents the vibrational force constants of the molecule and the vibrational frequencies of vibrational modes and the integrated intensities of infrared bands. Therefore, these calculations are valuable to provide insight into the vibrational spectrum. According to the quantum chemical literature [62], the B3LYP functional yields a good description of harmonic vibrational wavenumbers for small and medium sized molecules; the calculations refer to the isolated molecules while the vibrational spectra are recorded mostly in condensed phase. However, the quantum chemical results differ from the measured ones, consequently these calculations were scaled; in this sense, the thermal contributions to the vibrational energy were scaled by 0.963 [63].

#### 3.1.4. Chemical Shift Calculations

The calculations were performed to determine the ^1^H-, ^13^C- and ^11^B-NMR chemical shifts by the GIAO method [64,65], employing the B3LYP functional with the 6-311++G(d,p) basis set and using as references tetramethylsilane for ^1^H or ^13^C, and boron trifluoride for ^11^B. The solvent effect in the chemical shift was also calculated by using the self−consistent reaction field (SCRF) method and considering Tomasi’s polarizable continuum model (PCM) at the same theory level, using acetone as the medium. Finally, explicit interaction with the solvent was computed, in order to obtain the corresponding chemical shifts values [66,67].

#### 3.1.5. Reactivity Descriptors

The chemical potential (μ), hardness (η), nucleophilicity (*N*), electrophilicity (ω), Fukui fuction (*f*^−^),were calculated by the respectively equation [68,69,70,71,72]:

(1) $$\mu =-\frac{\left(I+EA\right)}{2}$$

(2) $$n=\frac{I-EA}{2}$$

(3) $$\omega =\frac{\mu^{2}}{2n}$$

(4) N=−I

(5) $$f_{k}^{-}=q_{k}\left(N\right)-q_{k}\left(N-1\right)$$

where *I* is the ionization energy and *EA* electron affinity, this values were calculated from the energies of the neutral, cationic and anionic forms of **I**–**III** at the same theory level. Additionnally, Parr functions (*P^−^*_k_, *P*^+^_k_), local nucleophilicity (*N*_k_) and local electrophilicity(ω_k_) indices of the phenilboronic carbons atoms were determined [34,38,73].

(6) $$P_{k}^{-}=\rho_{k}^{rc}$$

where ρ is the spin atomic density of cation radical specie.

(7) $$P_{k}^{+}=\rho_{k}^{ra}$$

where ρ is the spin atomic density of anion radical specie.

(8) $$N_{k}=NP_{k}^{-}$$

(9) $$\omega_{k}=\omega P_{k}^{+}$$

#### 3.1.6. Metabolism Prediction

The human metabolism of the studied molecules was studied using MetaPrint2D-Reaction, a xenobiotic metabolism prediction software based on data-mining and statistical analysis of known metabolic transformations reported in the scientific literature [35,37,74,75,76,77,78]. The corresponding results are displayed as circular coloured marks that predict the reactions at that site and the reaction types, to show the metabolite formed. The colour of the mark in the atoms indicates its normalized occurrence ratio (NOR). A high NOR indicates a more frequently reported metabolism site in the metabolite database.

### 3.2. Experimental Methods

Experimental Procedure for the Synthesis of Hybrid DIMs-Phenyl Boronic Acids Previously Reported in the paper: A Green Approach to the Production of Hybrid Diindolylmethane-Phenylboronic Acids via a 3MCR: Promising Antineoplasic Molecules [12].

## 4. Conclusions

The three regioisomers of some hybrid diindolylmethane-phenylboronic acids were studied by means of DFT. The structural, electronic and spectroscopic properties concurred with the experimental data reported for similar compounds. Furthermore, the existence of eight dimeric structures was proposed, some of them, comparable with experimental X-ray data from other works. In addition, the effect of the interactions of the target molecules with solvent in the ^1^H-NMR chemical shifts was evaluated and the results agree with the experimental data. The reactivity properties were determined for the studied compounds and correlated with the biological activity of these molecules. Finally, an approach to the metabolism of the evaluated compounds in humans was presented and correlated with the reactivity properties.

## Figures and Tables

**Table 1:** Conformational energy values for **I**–**III**.

Conformer	Isomers					
	I		II		III	
	Energy (Hartrees)	ΔE (kcal/mol)	Energy (Hartrees)	ΔE (kcal/mol)	Energy (Hartrees)	ΔE (kcal/mol)
*cis–trans (ct)*	−1173.13344187	0.0	−1173.13778578	0.0	−1173.13846019	0.0
*trans–trans (tt)*	−1173.13246585	0.7	−1173.13341930	2.7	−1173.13351019	3.1
*cis–cis (cc)*	−1173.12883432	3.3	−1173.13395050	2.4	−1173.13460054	2.4

**Table 2:** Relative energy difference between the conformers when the group –B(OH)_2_ is 90° of the benzene ring in the systems **I**: *ortho*, **II**: *meta* and **III**: *para*. In each case, the dihedral angle is between C12–C13–B–O.

Conformer	Isomers					
	I (90°)		II (90°)		III (90°)	
	Energy (Hartrees)	* ΔE (kcal/mol)	Energy (Hartrees)	* ΔE (kcal/mol)	Energy (Hartrees)	* ΔE (kcal/mol)
*cis–trans (ct)*	−1173.13188159	0.0	−1173.13317105	0.0	−1773.13273599	0.0
*trans–trans (tt)*	−1173.12910953	1.7	−1173.13082945	1.5	−1173.13071757	1.3
*cis–cis (cc)*	−1173.12453200	4.6	−1173.12514551	5.0	−1173.12527473	4.7

* Energies differences relative to more stable conformer: *ct*.

**Table 3:** Effect of the rotation of the boronic acid group on the conformational energy values of the structures for **I**–**III**.

Conformer						
Isomer	*cis-trans (ct)*		*trans-trans (tt)*		*cis-cis (cc)*	
	Energy (Hartrees)	ΔE (kcal/mol)	Energy (Hartrees)	ΔE (kcal/mol)	Energy (Hartrees)	ΔE (kcal/mol)
**I**	−1173.13344187	1.0	−1173.13246585	2.1	−1173.12883432	2.7
**I** (90°)	−1173.13188159		−1173.12910953		−1173.12453200	
**II**	−1173.13778578	2.9	−1173.13341930	1.6	−1173.13395050	5.5
**II** (90°)	−1173.13317105		−1173.13082945		−1173.12514551	
**III**	−1173.13846019	3.6	−1173.13351019	1.8	−1173.13460054	5.9
**III** (90°)	−1773.13273599		−1173.13071757		−1173.12527473	

**Table 4:** Selected theoretical and experimental bond length (Å) of the conformers for **I**, **II** and **III**.

Bond	I					II					III					Exp
	*ct*	*cc*	Δ*_cc-ct_*	*tt*	Δ*_tt-ct_*	*ct*	*cc*	Δ*_cc−ct_*	*tt*	Δ*_tt-ct_*	*ct*	*cc*	Δ*_cc−ct_*	*tt*	Δ*_tt−ct_*	
N1–H1	1.005	1.005	0.000	1.005	0.000	1.005	1.005	0.000	1.005	0.000	1.005	1.005	0.000	1.005	0.000	0.91 ^c^
O1–H(O1)	0.964	0.964	0.000	0.961	−0.003	0.963	0.960	−0.003	0.961	−0.002	0.963	0.960	−0.003	0.96	−0.003	0.92 ^b^
O2–H(O2)	0.960	0.960	0.000	0.961	0.001	0.960	0.960	0.000	0.961	0.001	0.960	0.960	0.000	0.96	0.000	0.92 ^b^
N1–C2	1.384	1.383	−0.001	1.384	0.000	1.382	1.382	0.000	1.383	0.001	1.383	1.383	0.000	1.383	0.000	1.388 ^a^
C2–C3	1.369	1.370	0.001	1.368	−0.001	1.369	1.370	0.001	1.369	0.000	1.370	1.370	0.000	1.37	0.000	1.366 ^c^
C3–C10	1.513	1.513	0.000	1.513	0.000	1.513	1.513	0.000	1.513	0.000	1.515	1.515	0.000	1.515	0.000	1.508 ^a^
N1′−C2′	1.384	1.381	−0.003	1.384	0.000	1.383	1.383	0.000	1.383	0.000	1.383	1.382	−0.001	1.383	0.000	1.379 ^b^
C2′−C3′	1.371	1.374	0.003	1.370	−0.001	1.370	1.370	0.000	1.370	0.000	1.369	1.370	0.001	1.369	0.000	1.370 ^c^
C3′−C10	1.515	1.517	0.002	1.515	0.000	1.515	1.515	0.000	1.515	0.000	1.513	1.513	0.000	1.513	0.000	1.513 ^b^
C10–C11	1.536	1.538	0.002	1.538	0.002	1.531	1.532	0.001	1.532	0.001	1.530	1.530	0.000	1.53	0.000	1.518 ^b^
C11–C12	1.415	1.414	−0.001	1.417	0.002	1.395	1.395	0.000	1.395	0.000	1.399	1.398	−0.001	1.399	0.000	1.372 ^b^
C12–C13	1.406	1.406	0.000	1.406	0.000	1.405	1.405	0.000	1.405	0.000	1.390	1.391	0.001	1.39	0.000	1.384 ^b^
C13–C14	1.392	1.392	0.000	1.391	−0.001	1.403	1.403	0.000	1.403	0.000	1.405	1.405	0.000	1.404	−0.001	1.385 ^b^
C14–C15	1.390	1.391	0.001	1.390	0.000	1.392	1.393	0.001	1.392	0.000	1.402	1.404	0.002	1.403	0.001	1.374 ^b^
C15–C16	1.394	1.393	−0.001	1.393	−0.001	1.392	1.392	0.000	1.393	0.001	1.392	1.393	0.001	1.392	0.000	1.374 ^b^
C16–C11	1.396	1.397	0.001	1.397	0.001	1.398	1.398	0.000	1.398	0.000	1.396	1.396	0.000	1.397	0.001	1.378 ^b^
C12–B	1.573	1.584	0.011	1.569	−0.004	-	-		-	-	-	-	-	-	-	1.579 ^b^
C13–B	-	-	-	-	-	1.565	1.575	0.010	1.559	−0.006	-	-	-	-	-	1.579 ^b^
C14–B	-	-	-	-	-	-	-		-	-	1.563	1.574	0.011	1.557	−0.006	1.579 ^b^
B–O1	1.367	1.372	0.005	1.374	0.007	1.368	1.368	0.000	1.375	0.007	1.368	1.368	0.000	1.376	0.008	1.355 ^b^
B–O2	1.375	1.362	−0.013	1.377	0.002	1.375	1.368	−0.007	1.376	0.001	1.375	1.369	−0.006	1.376	0.001	1.362 ^b^

^a^ Value obtained from Kumar et al., [19]; ^b^ Value obtained from Kurt [14]; ^c^ Value obtained from Maciejewska et al., [20] for 5,5-dicyano-3,3′-*bis*(diindolyl)methane.

**Table 5:** Selected theoretically and experimental bond angles (°) of the conformers of the target molecules.

Bond Angle	I					II					III					Exp
	*ct*	*cc*	Δ*_cc−ct_*	*tt*	Δ*_tt−ct_*	*ct*	*cc*	Δ*_cc−ct_*	*tt*	Δ*_tt−ct_*	*ct*	*cc*	Δ*_cc−ct_*	*tt*	Δ*_tt−ct_*	
C9–N1–C2	109.20	109.20	0.00	109.20	0.00	109.30	109.20	−0.10	109.20	−0.10	109.20	109.20	0.00	109.20	0.00	108.70 ^b^
C3–C10–C11	113.40	113.40	0.00	113.70	0.30	112.90	113.10	0.20	113.10	0.20	111.40	113.10	1.70	111.40	0.00	113.11 ^a^
C9′−N1′−C2′	109.20	109.30	0.10	109.20	0.00	109.30	109.20	−0.10	109.20	−0.10	109.20	109.20	0.00	109.20	0.00	108.80 ^b^
C3′−C10–C11	111.10	111.30	0.20	111.00	−0.10	111.60	111.50	−0.10	111.60	0.00	113.10	111.40	−1.70	113.10	0.00	110.60 ^a^
C11–C12–C13	118.20	118.00	−0.20	118.20	0.00	122.00	122.40	0.40	121.80	−0.20	121.00	120.90	−0.10	120.90	−0.10	119.20 ^b^
C12–C13–C14	122.10	122.10	0.00	122.10	0.00	117.90	117.60	−0.30	118.40	0.50	121.30	121.60	0.30	121.10	−0.20	123.00 ^b^
C14–C15–C16	119.90	119.80	−0.10	119.90	0.00	119.90	120.00	0.10	120.00	0.10	121.80	121.80	0.00	121.40	−0.40	123.00 ^b^
C15–C16–C11	121.50	121.40	−0.10	121.60	0.10	120.80	120.70	−0.10	120.90	0.10	121.50	120.70	−0.80	120.70	−0.80	119.40 ^b^
C11–C12–B	124.90	124.90	0.00	125.80	0.90	-	-	-	-	-	-	-	-	-	-	121.90 ^b^
B–C12–C13	116.90	117.10	0.20	116.00	−0.90	-	-	-	-	-	-	-	-	-	-	121.90 ^b^
C12–B–O1	121.30	124.30	3.00	121.10	−0.20	-	-	-	-	-	-	-	-	-	-	118.20 ^b^
C12–B–O2	121.90	120.70	−1.20	116.90	−5.00	−	-	-	-	-	-	-	-	-	-	122.20 ^b^
C12–C13–B	-	-	-	-	-	119.90	121.00	1.10	120.80	0.90	-	-	-	-	-	121.90 ^b^
B–C13–C14	-	-	-	-	-	122.20	121.40	−0.80	120.90	−1.30	-	-	-	-	-	122.70 ^b^
C13–B–O1	-	-	-	-	-	119.00	122.50	3.50	118.50	−0.50	-	-	-	-	-	118.20 ^b^
C13–B–O2	-	-	-	-	-	124.10	122.60	−1.50	118.40	−5.70	-	-	-	-	-	122.20 ^b^
C13–C14–B	-	-	-	-	-	-	-	-	-	-	120.20	121.60	1.40	121.20	1.00	121.90 ^b^
B–C14–C15	-	-	-	-	-	-	-	-	-	-	122.70	121.60	−1.10	121.20	−1.50	122.70 ^b^
C14–B–O1	-	-	-	-	-	-	-	-	-	-	118.80	122.60	3.80	118.40	−0.40	118.20 ^b^
C14–B–O2	-	-	-	-	-	-	-	-	-	-	124.30	122.50	−1.80	118.50	−5.80	122.20 ^b^
B–O1–H(O1)	112.10	113.60	1.50	116.10	4.00	112.10	113.30	1.20	116.20	4.10	112.20	113.40	1.20	116.20	4.00	111.40 ^b^
B–O2–H(O2)	113.70	113.00	−0.70	115.80	2.10	114.40	113.40	−1.00	116.10	1.70	114.60	113.30	−1.30	116.20	1.60	115.60 ^b^
O1–B–O2	116.70	114.90	−1.80	122.00	5.30	116.90	114.90	−2.00	126.10	9.20	116.90	114.80	−2.10	123.10	6.20	119.50 ^b^

^a^ Value obtained from Kumar et al., [19]; ^b^ Value obtained from Maciejewska et al., [20] for 5,5-dicyano-3,3′-*bis*(diindolyl)methane.

**Table 6:** Energetic properties of the eight dimers found.

Dimer	Electronic Energy (Hartrees)	IE (kcal/mol)	RE (kcal/mol)
**III**-1	−2346.28916998	−7.7	0.0
**III**-2	−2346.28480554	−4.9	2.7
**III**-3	−2346.28456653	−4.8	2.9
**III**-4	−2346.28343557	−4.1	3.6
**III**-5	−2346.28249966	−3.5	4.2
**III**-6	−2346.28131638	−2.8	4.9
**III**-7	−2346.28044096	−2.2	5.5
**III**-8	−2346.27761899	−0.4	7.3

IE = interaction energy; RE = Relative Energy.

**Table 7:** Values of electronic density and the square Laplacian of density for the dimers.

Dimer	Interaction	*ρ*	∇^2^*ρ*	Dimer	Interaction	*ρ*	∇^2^*ρ*
1	BCP (3,−1) O1H●●●O1H	0.0289	−0.0264	5	BCP (3,−1) N–H1●●●π indol	0.0063	−0.0045
	BCP (3,−1) O2H●●●O2H	0.0293	−0.0267		BCP (3,−1) C–H2●●●π	0.0034	−0.0027
	RCP (3,+1)	0.0043	−0.0053		RCP (3,+1)	0.0034	−0.0028
2	BCP (3,−1) O1H●●●NH1	0.0165	−0.0149	6	BCP (3,−1) O1H●●●N	0.0110	−0.0083
	BCP (3,−1) CH2 indol●●●π boronic	0.0037	−0.0026				
	RCP (3,+1) C–H2●●●π O1H●●●NH1	0.0023	−0.0023		BCP (3,−1) NH1●●●O2	0.0202	−0.0191
	BCP (3,−1) N●●●H2–C indol	0.0036	−0.0027				
	BCP (3,−1) N–H1π●●●indol	0.0038	−0.0029		RCP (3,+1)	0.0075	−0.0081
	RCP (3,+1) N●●●H–C indol	0.0026	−0.0024				
	N–H1●●●π indol						
3	BCP (3,−1) O1H●●●O1	0.0223	−0.0211	7	BCP (3,−1) O1H●●●π indol	0.0047	−0.0030
	RCP (3,−1) O1H●●●O2	0.0231	−0.0226		BCP (3,−1) C–H2●●●π	0.0042	−0.0032
	RCP(3,+1)	0.0098	−0.0121		BCP (3,−1) CH●●●O	0.0027	−0.0022
					RCP (3,+1) CH●●●π OH●●●π	0.0027	−0.0022
					RCP (3,+1) OH●●●π CH●●●O	0.0010	−0.0009
4	BCP (3,−1) NH1●●●π indol ^1^	0.0057	−0.0039	8	BCP (3,−1) C–H2●●●O2	0.0033	−0.0031
	BCP (3,−1) C–H2●●●π indol ^1^	0.0033	−0.0023		BCP (3,−1) O1H●●●π indol	0.0097	−0.0066
	BCP (3,−1) NH1●●●π indol ^2^	0.0063	−0.0045		RCP (3,+1)	0.0031	−0.0031
	BCP (3,−1) C–H2●●●π indol ^2^	0.0034	−0.0027				
	RCP (3,+1) ^1^	0.0031	−0.0025				
	RCP (3,+1) ^2^	0.0034	−0.0028				

*ρ* = electron density (e/a.u.^3^); ∇^2^*ρ* = Laplacian of density; ^1^ and ^2^ Indole ring of indolylic moiety.

**Table 8:** Experimental and theoretical infrared frequencies for the conformer *ct* to **I**–**III** and their approximate mode description.

Experimental Frequencies			Theoretically Results									Mode Descriptions
I	II	III	*ct*-I			*ct*-II			*ct*-III			
			F	S ^a^	S ^b^	F	S ^a^	S ^b^	F	S ^a^	S ^b^	
3405.1	3403.5	3407.1	3875.5	3720.5	3712.7	3881.4	3726.1	3718.3	3882.9	3727.6	3719.8	υO2H
			3832.4	3679.1	3671.4	3845.5	3691.7	3684.0	3847.5	3693,6	3685.9	υO1H
			3674.9	3527.9	3520.6	3674.8	3527.8	3520.4	3674.1	3527.2	3519.8	υN1’H
			3674.1	3527.2	3519.8	3674.4	3527.5	3520.1	3673.6	3526.7	3519.3	υN1H
			3189.0	3061.5	3055.1	3188.7	3061.2	3054.8	3189.4	3061.8	3055.5	υsC_4_H + υsC_5_H + υsC_6_H + υsC_7_H
3043.0	3077.9	3080.0	3188.7	3061.2	3054.8	3188.1	3060.6	3054.2	3189.1	3061.5	3055.2	υsC_4’_H + υsC_5’_H + νsC_6’_H + υsC_7’_H
			3186.3	3058.9	3052.5	3182.9	3055,5	3049.2	3182.8	3055.5	3049.1	υsC_16_H + υsC_15_ + υsC_13_ + υsC_12_
2949.5	3000.0	2991.5	3179.9	3052.7	3046.3	3178.8	3051.7	3045.3	3179.7	3052.5	3046.2	υasC_4_H + υasC_6_H + υasC_7_H
			3179.2	3052.1	3045.7	3177.8	3050.7	3044.3	3178.8	3051.6	3045.3	υasC_4’_H + υasC_6’_H + υasC_7’_H
2885.9	2962.7	2943.6	3177.5	3050.4	3044.1	3176.1	3049,0	3042.7	3177.7	3050.5	3044.2	υasC_16_ + υasC_14_ + υasC_15_ + υasC_13_
			1656.2	1623.1	1628.1	1656.5	1623.3	1628.3	1656.7	1623.6	1628.5	υasIndolyl
1731.0	1730.9	1720.7	1655.6	1622.5	1627.4	1656.0	1622.9	1627.8	1656.1	1623.0	1628.0	υasIndolyil’
			1634.6	1601.9	1606.8	1637.0	1604.2	1609.1	1645.5	1612.6	1617.5	υasbenzene ring
			1614.0	1581.8	1586.6	1613.5	1581.2	1586.1	1613.8	1581.5	1586.4	υasIndolyl + υasIndolyl’
			1612.2	1579.9	1584.8	1611.1	1578.9	1583.7	1611.7	1579.4	1584.3	υasIndolyl + υasIndolyl’ + υasbenzene ring
1597.3	1601.7	1607.9	1519.9	1489.5	1494.0	1583.9	1552.2	1557.0	1584.3	1552.6	1557.4	υasIndolyl + υasIndolyl’ + δN_1_H + δN_1’_H
			1519.3	1488.9	1493.4	1520.0	1489.6	1494.2	1579.2	1547.7	1552.4	υasIndolyl + υasIndolyl’ + δN_1_H + δN_1’_H + δC_5_H + δC_5’_H_’_
1456.6	1455.9	1456.3	1508.0	1477.9	1512.6	1479.2	1449.6	1425.0	1443.8	1415.0	1419.3	υsC_11_C_12_C_15_C_16_ + δH_13_H_15_
			1480.5	1450.9	1426.1	1452.4	1423.3	1399.2	1442.8	1414.0	1418.3	υasC_8_C_9_C_4_C_5_C_6_C_7_ + δH_4_H_5_
1438.6	1419.2	1408.5	1469.4	1440.0	1415.5	1444.3	1415.4	1391.4	1433.1	1404.5	1408.8	υasC_11_C_12_C_13_C_14_C_15_C_16_ + δH_14_H_15_H_16_
			1388.9	1361.1	1337.9	1442.3	1413.5	1389.5				δIndolyl + δIndolyl’ + δbenzene ring
1370.6		1392.0	1381.9	1354.3	1331.2	1385.9	1358.2	1362.3	1519.5	1489.1	1493.6	δIndolyl + δIndolyl’ + δbenzene ring + υasBO1 + δoopC10H

S ^a^ = Frequencies scaled with correction factors: below of 1700 cm^−1^ factor of 0.98 and the rest of the frequencies with 0.96; S ^b^ = Frequencies scaled with correction factors: in range of 1700–4000 cm^−1^ with factor of 0.958, and below with 0.983; υ = stretching; υs = symmetric stretching; υas = asymmetric stretching; δ = in the plane bending; γ = out of plane bending.

**Table 9:** Reactivity properties of objective molecules and the corresponding relationship with their biological effect.

Property		*ct*-I	*ct*-II	*ct*-III
Energy (Hartree)	Neutral	−1173.13344187	−1173.13778578	−1173.13846019
	Negative	−1173.12670369	−1173.13148373	−1173.13365972
	Positive	−1172.88003603	−1172.88224063	−1172.87999701
Reactivity (Ev)	*EA*	−0.18	−0.17	−0.13
	I	6.90	6.95	7.03
	µ	−6.80	−6.87	−6.97
	η	3.54	3.56	3.58
	ω	6.54	6.62	6.78
	*N*	−6.90	−6.95	−7.03
Biological activity (%)	U251	36	49	7.59
	PC-3	62.79	50.63	26.85
	K562	0.00	19.42	0.00
	HCT-15	47.59	59.13	15.96
	MCF-7	60.27	81.81	48.40
	SKLU-1	44.11	63.99	11.09

Neutral = neutral specie; Positive = cationic specie; Negative = anionic specie; *EA* = electronic affinity; *I* = ionization energy; μ = chemical potential; η = hardness; ω = electrophilicity; *N* = nucleophilicity index [34]; U251 = glia of central nerve system cancer cells; PC-3 = prostate cancer cells; K562 = leukemia cancer cells; HCT-15 = colon cancer cells; MCF-7 = breast; SKLU-1 = lung cancer cells.

**Table 10:** Selected local reactivity parameters of **I**–**III**.

Atom	*f*^−^			*P*^−^_k_			*P*^+^_k_			*N*_k_			ω_k_		
	I	II	III	I	II	III	I	II	III	I	II	III	I	II	III
C11	0.030	0.020	0.060	0.010	0.030	0.010	0.020	−0.040	0.010	−0.140	0.280	−0.070	0.050	0.220	0.030
C12	−0.010	0.080	0.010	0.000	−0.010	0.000	0.010	0.010	0.000	−0.030	−0.050	0.020	0.010	−0.070	0.030
C13	−0.010	−0.170	0.010	−0.010	0.010	0.000	0.020	0.000	0.000	−0.140	−0.030	−0.010	−0.050	0.040	0.020
C14	−0.030	0.030	0.070	0.010	0.010	0.000	0.030	0.000	−0.060	−0.210	−0.010	0.390	0.040	0.090	−0.030
C15	−0.010	0.230	−0.030	0.020	−0.010	−0.010	0.040	−0.030	0.000	−0.280	0.210	−0.010	0.130	−0.030	−0.040
C16	0.000	−0.040	0.020	0.040	0.010	0.000	0.020	0.060	−0.010	−0.140	−0.420	0.040	0.260	0.030	−0.010
